# CRP to Albumin Ratio as a Prognostic Nutrition-Based Biomarker for Patients With Gastric Cancer: A Narrative Review

**DOI:** 10.7759/cureus.71516

**Published:** 2024-10-15

**Authors:** Grigorios Christodoulidis, Alexandros Voutyras, George Fotakopoulos, Konstantinos-Eleftherios Koumarelas, Vasiliki E Georgakopoulou, Marina-Nektaria Kouliou, Eirini Sara Agko, Kyriaki Tsagkidou, Dimitra Bartzi, Iraklis Kagkouras, Dimitrios Zacharoulis

**Affiliations:** 1 Surgery, General University Hospital of Larissa, Larissa, GRC; 2 Neurosurgery, Aristotle University of Thessaloniki, Thessaloniki, GRC; 3 Emergency Medicine, General University Hospital of Larissa, Larissa, GRC; 4 Pathophysiology and Pulmonology, Laiko General Hospital, Athens, GRC; 5 Intensive Care Unit, Asklepios Paulinen Klinik Wiesbaden, Wiesbaden, DEU; 6 Gastroenterology, General University Hospital of Larissa, Larissa, GRC; 7 Oncology, 251 Air Force General Hospital, Athens, GRC; 8 Surgery, Worcestershire Acute Hospitals NHS Trust, Worcester, GBR

**Keywords:** c-reactive protein to albumin ratio, crp/alb, gastric cancer, prognostic biomarker, stomach cancer

## Abstract

Gastric cancer stands as a significant global health challenge, ranking among the top malignancies worldwide in terms of prevalence and mortality. Despite advances in treatment modalities, including surgical intervention and chemotherapy, its prognosis remains largely unfavorable, with late-stage diagnoses contributing to high mortality rates. In recent years, attention has turned to inflammation-based prognostic markers, notably the CRP to albumin ratio (CAR), as potential indicators of disease progression and patient outcomes postoperatively. The CAR index is observed to have the potential as an independent predictor of survival outcomes, including overall survival (OS), disease-free survival, recurrence-free survival, and cancer-specific survival, but with inconsistent data. Discrepancies in defining optimal CAR cutoff values have been observed, and there are no standardized criteria. CAR is also integrated into the bibliography with other prognostic factors for the development of novel prognostic indices that offer avenues for refining risk stratification and enhancing patient care in gastric cancer management. In this narrative review, we study the utility of the CAR index for the OS of gastric cancer patients after a curative or palliative gastrectomy.

## Introduction and background

Gastric cancer stands out as the fifth most prevalent malignancy [1-5], comprising 5.7-8% of global cancer incidences [6,7]. It ranks as the second and fifth most common malignancy in males and females, respectively, with an annual diagnosis of approximately one million new cases [3,8,9]. Gastric cancer contributes to about 10% of cancer-related deaths, totaling around 800,000 fatalities annually [3,6,7].

These figures position gastric cancer as the second leading cause of gastrointestinal malignancy-related mortality and the third cause of cancer-related mortality [2-4,8,10]. The elevated mortality rates are frequently associated with late-onset symptoms, resulting in delayed diagnoses [11]. Surgical intervention, with or without chemotherapy, remains the pivotal management strategy, notwithstanding postoperative complications reaching rates as high as 28% after laparoscopic gastrectomy [12]. Despite improvements, recurrence rates can still reach up to 45.5% [13,14].

The five-year survival rate exhibits substantial variability, ranging from 10% to 30%, underscoring the necessity for additional surgical resection factors, given the disparate outcomes between early and advanced cases [10,15,16]. The tumor-node-metastasis (TNM) classification - introduced by the International Union Against Cancer (UICC) and the American Joint Committee on Cancer (AJCC) guidelines - founded on tumor characteristics, invasion, lymph node metastasis, and distant metastasis, persists as a dependable prognostic indicator [4,17]. Nonetheless, the heterogeneous nature of the disease mandates the development of novel markers with heightened specificity and sensitivity [3,16].

Recent strides in histochemical and molecular factors have precipitated the emergence of malignancy prognostic factors, encompassing both tumor-specific and host-related elements. Inflammation-based and nutritional markers, including CRP to albumin ratio (CAR), neutrophil to lymphocyte ratio (NLR), platelet to lymphocyte ratio (PLR), prognostic nutritional index (PNI), Glasgow Prognostic Score (GPS), and Prognostic Index (PI), present a cost-effective avenue [2,4]. CAR, a groundbreaking inflammation-based prognostic indicator defined as the CRP to albumin ratio, serves as a reflection of both inflammatory and nutritional status, surpassing conventional inflammatory indexes [1,4,5].

Elevated CAR values align with an exacerbated general condition, poorer outcomes in afflictions like sepsis, pancreatitis, and cancer, alongside diminished overall survival (OS), recurrence-free survival (RFS), and cancer-specific survival (CSS) in gastric cancer. CAR outshines traditional inflammatory indexes by offering insights into the combined inflammatory and nutritional status of cancer patients [1,4,5,16,18]. CRP, a hepatic-synthesized acute phase response protein indicating systemic inflammation, plays a pivotal role in shaping the tumor microenvironment. Preoperative inflammation establishes links with tumor metastasis, invasion, chemotherapy resistance, DNA damage, promotion of angiogenesis, and inhibition of apoptosis [12,19].

Albumin, also synthesized by the liver, represents the principal constituent of human serum total protein and plays a pivotal role in maintaining nutritional equilibrium. Tumor patients frequently exhibit diminished serum albumin levels due to compromised nutritional status [16,20-22]. Consequently, CAR stands as a superior metric to traditional inflammatory indexes, reflecting the combined inflammatory and nutritional status of cancer patients [1,4,5]. Elevated CAR values signify a worsened general condition in tumor patients, poorer outcomes across various diseases, and an inferior OS, RFS, and CSS in gastric cancer [16,18,23]. Despite the unclear clinical impact of CAR on both short- and long-term oncologic outcomes for gastric cancer patients, this narrative review aspires to evaluate the role of CAR in predicting the survival of patients undergoing curative-intent surgery for gastric adenocarcinoma [16,18,23].

## Review

Methods

A comprehensive literature search was systematically conducted across PubMed, Embase, Web of Knowledge, Cochrane Library, and Google Scholar to identify articles comparing OS, RFS, CSS, and prognosis among patients with gastric cancer following surgical resection, contingent upon the preoperative CAR. The search utilized a combination of keywords, including “CAR,” “CRP to Albumin ratio,” “CRP/Albumin,” “GC,” “Gastric Cancer,” “Gastric Adenocarcinoma,” “Gastroesophageal Cancer,” and “Survival.” These terms were employed both independently and in various logical combinations using “OR” and “AND” to ensure the retrieval of the maximal number of relevant articles.

Exclusion criteria involved eliminating repetitive and non-English studies. Following an initial screening of titles and abstracts, a meticulous review of the full-text copies of identified articles was undertaken. In instances of any disagreements encountered throughout the screening and review phases, resolution was achieved through consultation with a senior reviewer.

The selection process is presented in Table 1.

**Table 1:** Study selection process

Phase	Number of records (n)
Records identified	320
Records after duplicates removed	290
Records screened (titles and abstracts)	290
Exclusions (non-English, repetitive studies)	150
Full-text articles assessed	140
Further exclusions	132
Articles included in the review	8

Results

We identified eight studies with non-metastatic gastric cancer that received curative surgery and four studies in which the patients received both curative and palliative surgery between 2015 and 2023. In these studies, researchers reviewed the prognostic value of preoperative CAR in terms of survival. A comprehensive evaluation of multiple studies provides an insightful understanding of the prognostic value of CAR in gastric cancer patients. Xu et al. established that a CAR cutoff of 0.131 serves as a significant predictor of early recurrence following gastrectomy [13]. However, Toyokawa et al. observed that a CAR cutoff of 0.03 signifies a significant decrease in survival rates within the high-CAR group (64.7%) compared to the low-CAR group (85.5%). In the multivariate analyses, CAR was found to be an independent prognostic factor for OS with an HR of 1.707 and a 95% CI of 1.016-2.867 (p = 0.044) [4].

These findings were also supported by Liu et al., who reported an optimal cutoff for the CAR as 0.025 for OS, with considerably elevated survival rates among patients with values below this threshold [24]. This leads to a sensitivity of 76.8% and a specificity of 48.1%. Patients were divided into two groups based on this cutoff value. The one-, three-, and five-year OS rates in patients with a CAR less than or equal to 0.025 were 88.3%, 60.7%, and 54.5%, respectively. These survival rates were significantly higher than those in patients with a CAR greater than 0.025, which were 76.9%, 32.6%, and 26.9%, respectively. In addition, when stratified by the TNM stage, the prognostic significance of the CAR ratio was still maintained in stages I to III. A low CAR was associated with a longer median OS in all these stages [24]. Research conducted by Aoyama et al. further identified higher OS rates at three and five years post-surgery for the low-CAR group (92.5% and 87.9%, respectively) compared to the high-CAR group (84.9% and 71.9%, respectively) [23].

Kudou et al. divided their patients into two categories based on tumor location: adenocarcinoma of the esophagogastric junction (AEG) and upper gastric cancer (UGC) [2]. The patients’ OS was studied in relation to various inflammation-based prognostic scores. In a similar vein, the cutoff value for CAR was 0.1, and patients with CAR ≥0.1 had poorer five-year RFS and OS rates. Those with a CAR ≥0.1 had an RFS rate of 44.9% vs. 77.9% and an OS rate of 43.4% vs. 82.0%. In multivariate analyses, it was found that the N-stage and CAR ≥0.1 were independent predictive factors for OS in patients with AEG and UGC [25]. However, the two-institute study by Kudou et al., published in 2019, indicated that the CAR index, with an optimal cutoff value of 0.017, had a prognostic value only in the univariate analysis and not in the multivariate [2]. This was echoed in the study by Li et al., who concluded that higher preoperative and postoperative CAR were linked to shorter OS [26]. Aoyama et al. found an association between high CAR values (over the median of 0.022) and reduced survival times [23]. The CAR was found to perform well in predicting one-, three-, and five-year survival in patients with gastric cancer, with area under the curve values of 0.758, 0.742, and 0.787, respectively. However, when patients were divided into clinical stage subgroups (stage I, stage II + III, and stage IV), CAR was not associated with the prognosis of patients in these subgroups (p > 0.05). The study also found a significant increase in CAR value in patients with the M1 stage compared with the M0 stage (p < 0.001) and in stage IV compared with stage I and stage II + III (p = 0.001). These indicate that the prognostic value of CAR is in accordance with TNM staging [18]. Saito et al. observed significant differences in 5-year survival rates between high and low CAR groups (81.7% vs. 90.3%, respectively) [6]. Mao et al. reported that a high CAR was correlated with a significantly decreased OS rate [11].

In terms of disease-free survival (DFS), Gu et al. established that CAR was significantly associated with the five-year DFS rate, with a cutoff value of 0.05 [17]. Li et al. determined that higher preoperative CAR (cutoff 0.08) and higher postoperative CAR (cutoff 0.13) were linked to poorer DFS [26]. In the sphere of disease-specific survival, Liu et al. reported that an elevated CAR (greater than 0.2) was correlated with shorter CSS [8].

Regarding RFS, Aoyama et al. reported that RFS rates at three- and five-year post-surgery were notably higher for the low-CAR group (89.1% and 85.5%, respectively) compared to the high-CAR group (81.0% and 72.2%, respectively) [23]. Kudou et al. found that patients with a CAR equal to or greater than 0.1 exhibited poorer five-year RFS rates [25].

The CAR was also found to be an independent predictive factor for early recurrence with an HR of 2.108 from multivariate analysis of Xu et al. [13]. Early recurrence was associated with preoperative CAR ≥0.131. In patients with stage III gastric cancer, the CAR value influenced the effect of adjuvant chemotherapy on early recurrence. For patients with a CAR less than the optimal cutoff of 0.131, the risk of early recurrence was significantly lower for those who received one to three cycles (17.3% vs. 50.0%, p = 0.029) or more than three cycles of postoperative adjuvant chemotherapy (4.2% vs. 50.0%, p < 0.001) than those who did not receive chemotherapy. For patients with a CAR greater than 0.131, more than three cycles of chemotherapy significantly reduced the risk of early recurrence compared to those without chemotherapy (16.0% vs. 54.2%, p = 0.004). However, the early recurrence rate was similar between patients receiving one to three cycles and those without chemotherapy (58.3% vs. 54.2%, p = 0.824); thus, postoperative chemotherapy with one to three cycles is not beneficial to these patients [13].

The CAR was found to be significantly associated with other variables such as age, tumor location, metastatic lymph node ratio, and TNM stage. The multivariate analysis indicated that the CAR was an independent prognostic factor for OS [24]. On comparing the patient backgrounds between the high-CAR (CAR ≥0.05) and low-CAR (CAR<0.05) groups, Aoyama et al. found the same associations with significant differences noted in the pathological T status, N status, and lymphovascular invasion in the high-CAR group [23]. Patients from Kudous et al.’s retrospective study with a CAR ≥ 0.1 were observed to have more advanced cancer stages [11,25]. Furthermore, the CAR was significantly higher in patients older than 67 years, with male sex, and with indicators of tumor progression such as tumors >30 mm, lymph-node metastasis, poor differentiation, and high carcinoembryonic antigen level as proposed by Toiyama et al. [7].

Discussion

The role of inflammation in cancer development and progression is significant [1]. The systemic inflammatory response is responsible for a series of events leading to the transformation of normal cells into malignant ones, their survival, proliferation, invasion, and dissemination [1,4,9]. Inflammatory cells such as neutrophils, macrophages, and certain types of lymphocytes contribute critically to the tumor microenvironment and can paradoxically promote and inhibit tumor growth. CRP is a highly sensitive marker for inflammation and serves as a prognostic indicator in various primary malignancies, including gastric cancer [12,19]. CRP is primarily produced by liver cells in response to proinflammatory cytokines such as tumor necrosis factor α and IL-6 [19]. These cytokines stimulate angiogenesis, thereby enhancing the progression and metastasis of malignant tumors [12,19].

Serum albumin, a conventional biomarker for assessing a patient’s nutritional status, is produced by the liver and plays a crucial role in maintaining oncotic pressure, transporting various substances, and exhibiting immunomodulatory activity [16,20-22]. Lower levels of serum albumin, indicative of a malnourished state, have been associated with poorer survival outcomes in various types of cancers. Serum markers, including various proteins and metabolites, are valuable for diagnosing cancer, predicting patient survival rates, and monitoring disease recurrence following surgical treatment [20-22]. Several pretreatment serum-based inflammatory indicators like the NLR, the modified GPS, PLR, and CRP can be derived from routine blood tests and have been associated with prognosis in various types of cancer [16,18,23].

The discussed studies provide insightful data on the role of inflammation-based markers, particularly the CAR, in predicting postoperative complications and survival outcomes in gastric cancer patients [16,18,23]. With the advances in science, new biomarkers with increased potential arise.

Toyokawa et al. constructed a new PI, the CAR-PNI score, which emerged as an independent factor for OS [4]. The high-CAR group showed a significantly higher frequency of hematogenous recurrence and a higher rate of death from the primary disease [4]. In Toyokawa et al.’s study, the CAR-PNI score was found to be a significant prognostic indicator for stage II gastric cancer patients [4]. The five-year survival rates and recurrence patterns varied significantly based on the score. For example, the five-year OS rates were 94.6% for a CAR-PNI score of 0, 77.1% for a score of 1, and 58.0% for a score of 2 [4].

Xu et al. showed that integrating the CAR value with the pTNM stage significantly enhanced the model’s prognostic predictive performance [13]. The combined model had a lower Akaike information criterion of 456.6 and a Bayesian information criterion of 464.6, indicating a better fit [13]. The concordance index was also higher at 0.82 for the combined model, showing a significant improvement in prediction (p < 0.001) [13].

In the study by Liu et al., a new systemic prognostic score (SPS) was developed based on the CAR index, PNI, preoperative body weight loss, and CA 19-9 markers [8]. The SPS, which ranged from 0 to 4, was shown to be an independent predictor of CSS, alongside tumor location and TNM stage [8]. The five-year CSS rates were observed to decrease with higher SPS scores: 67.2% for SPS 0, declining to 10.6% for SPS 3/4. This suggests a clear inverse relationship between the SPS and patient survival outcomes [8].

Pancreatic fistula and abdominal abscess were observed by Aoyama et al. as high as 8% (8/100) and 4% (4/100), respectively, in the high-CAR group but 3.2% (12/381) and 1.0% (4/381), respectively, in the low-CAR group [23]. Adjuvant treatment refusal was considerably greater at 29.7% (11/37) in the high-CAR group compared with 14.6% (13/89) in the low-CAR group [23].

Saito et al. introduced and investigated the combination of the CAR and the NLR as an independent prognostic predictor [6]. Of the 453 patients, 95 were both CARHigh and NLRHigh, with a five-year survival rate of 59.6%; 179 were either CARHigh or NLRHigh, with a five-year survival rate of 75.8%; and 179 were both CARLow and NLRLow, with a five-year survival rate of 87.5% [6].

Similar results were found in Mao et al., with the CAR and NLR classifications being independent prognostic factors [11]. The study classified patients into subgroups according to CAR (<0.38/≥0.38) and NLR (<3.41/≥3.41) [11]. The median OS values for patients in groups I, II, and III were 5.10, 17.80, and 46.50 months, respectively, with group III being the patients with high CAR and NLR [11].

Another study evaluated the prognostic significance of CAR and NLR in patients with unresectable advanced gastric cancer, revealing a positive correlation between CAR and NLR. Patients with lower CAR and NLR values had significantly higher median survival times [27].

Additionally, a multi-institutional retrospective cohort study of 278 patients treated with nivolumab for gastric cancer assessed the impact of laboratory findings, immune-related adverse events (irAEs), and clinicopathological factors on long-term survival [28]. The occurrence of irAEs, high albumin levels, high lymphocyte count, and differentiated histological type were associated with improved survival [28]. This study highlights the potential of CAR, NLR, and other factors as prognostic markers in advanced gastric cancer treatment [28].

CAR is proved to be an independent prognostic factor for the OS not only of gastric patients but of esophageal cancer patients [29]. Sakai et al. revealed that patients exhibiting a CAR value equal to or greater than 0.026 had a significantly lower OS rate when compared to those with CAR values less than 0.026 (p < 0.001) [29].

The cutoff value of the CRP ratio is not fixed, and as a result, each researcher defines a different value according to their findings [12]. In the study of Tanaka et al., a cutoff value of 2.13 offered 55% sensitivity and 82% specificity for postoperative severe complications, representing the optimal cutoff [12]. The negative predictive value for postoperative complications was 94% for those with a low CRP ratio (<2.1), which comprised 80% (357 out of 449) of the patient [12].

Sun et al. concluded that postoperative CAR might be a useful predictor for postoperative complications after gastrectomy for gastric cancer [9]. When patients were divided into two groups based on the CAR cutoff value, those with a high CAR (≥3.04) were more likely to have postoperative complications than those with a low CAR (<3.04), 44.5% versus 15.8%, respectively (p < 0.001) [9].

Gülmez’s study showed a significant correlation between an elevated CAR and the prediction of postoperative infectious complications [10]. Specifically, the study found that a CAR above 1.77 could predict postoperative infectious complications with a sensitivity of 31.1% and a specificity of 88.8% [10].

Ai et al. studied a different index and demonstrated that ΔALB could be an independent risk factor for normal preoperative serum albumin patients to indicate short-term postoperative complications [20]. Patients with high ΔALB showed a higher risk of complications compared with patients with low ΔALB (62.3 versus 13.7%) [20].

Another novel index, the alkaline phosphatase to albumin ratio, is an independent prognostic factor for gastric cancer, and when combined with cancer staging, it can provide a more accurate assessment of patient prognosis [30].

An elevated CAR index in patients may require close observation and more intensive care after gastrectomy, with the patients benefiting from anti-inflammatory therapy or nutritional support [31].

## Conclusions

The CAR index serves as an independent predictor for various survival outcomes, including OS, disease-free survival, RFS, and CSS. The varying CAR cutoff values proposed in the bibliography highlight the need for personalized thresholds based on patient characteristics or the need for establishing a widely accepted cutoff value. CAR can also be integrated with other prognostic factors, such as the neutrophil-lymphocyte ratio and nutritional status, enhancing its predictive accuracy. Additionally, the development of novel prognostic indices, like the CAR-PNI score and SPS, further refines risk stratification. Further research into CAR’s optimal utilization and combination with other biomarkers holds promise for enhancing prognostication and patient care in gastric cancer management.
